# Sir Benjamin Collins Brodie

**Published:** 1899-06

**Authors:** 


**Sir Benjamin Collins Brodie.** By TIMOTHY HOLMES. Pp. 256. London: T. Fisher Unwin. 1898.

Mr. Holmes has produced one of the most interesting books yet issued in the "Masters of Medicine" series. It does not perhaps quite equal in interest the *Life of John Hunter*, but it does not fall far short of it. Mr. Holmes thinks that Brodie is to be placed next to Hunter among the celebrated surgeons of the past, and his well-known books, *Diseases of the Urinary Organs* and *Diseases of the Joints* are sufficient to justify this estimate of his work.

We cannot attempt in this short review to give even a brief outline of the events which make this volume so well worthy of perusal. Brodie's character as a man deserves very careful study; and the insight which the book gives us into the condition of the profession and the state of society at the time when he lived is by no means its least instructive part.

Brodie had many interests outside his profession, and his views on various questions of psychology are given by Mr. Holmes. Perhaps the keynote of Brodie's life and work may be found in his statement that "there is no greater happiness in life than that of surmounting difficulties; and nothing will conduce more than this to improve your intellectual faculties, or to make you satisfied with the situation which you have attained in life, whatever it may be." The book will therefore interest the general reader.

The frontispiece is a very beautiful reproduction of a photograph of the portrait of Brodie, painted by Watts in 1860. The photograph was taken by his grandson, the present Sir Benjamin Brodie, to whom the reader is also indebted for much of the information relating to the home-life of the distinguished surgeon.

